# Metabolite profile changes and increased antioxidative and antiinflammatory activities of mixed vegetables after fermentation by *Lactobacillus plantarum*

**DOI:** 10.1371/journal.pone.0217180

**Published:** 2019-05-22

**Authors:** Jungyeon Kim, Kum-Boo Choi, Ju Hun Park, Kyoung Heon Kim

**Affiliations:** 1 Department of Biotechnology, Graduate School, Korea University, Seoul, South Korea; 2 Pulmuone Institute of Technology, Pulmuone, Seoul, South Korea; Kyungpook National University, REPUBLIC OF KOREA

## Abstract

Fermented vegetables have emerged as prebiotics with various health benefits. However, the possible mechanisms behind their health benefits are unclear. To relate the metabolite profile changes in fermented mixed vegetables with associated health benefits of fermented vegetables, we analyzed the metabolite profiles of mixed vegetables, before and after fermentation by *Lactobacillus plantarum*, using gas chromatography/time-of-flight–mass spectrometry (GC/TOF–MS). To analyze health benefits of fermented vegetables, antioxidative and antiinflammatory activities were measured using RAW 264.7 cells. Among 78 metabolites identified by GC/TOF–MS in this study, those significantly increased after fermentation include antioxidative and/or antiinflammatory agents such as lactate, 3-phennyllactate, indole-3-lactate, β-hydroxybutyrate, γ-aminobutyrate, and glycerol. These metabolites may have been either newly synthesized or depolymerized from high molecular weight polymers from vegetables during fermentation. This is the first metabolomics study to relate metabolite profile changes with increased health benefits of fermented vegetables.

## Introduction

The global market of functional foods, which are known to induce health benefits and help cure diseases, is rapidly growing [1,2]. The functional food market accounts for 5% of the overall food market and contributes greatly to the growth of the food industry [1,3]. Among various functional food markets, the probiotic food market is most active, accounting for 60–70% of the entire functional food market [4]. The production and consumption of non-dairy probiotic food, such as probiotic-fermented vegetables, have been especially growing due to the ongoing trend of vegetarianism and global prevalence of lactose intolerance [2,5].

Generally, probiotics require prebiotics to survive in gastric environment [5]. Vegetables are also recognized as prebiotics that provide nutrition to probiotics, thus resulting in health benefits on gastrointestinal environment [5]. Likewise, vegetables fermented with probiotic starters strongly enhance the production of beneficial molecules which inhibit the growth of pathogens in gastrointestinal environment, hence offering health benefits in the form of their antioxidative and antiinflammatory effects [5,6]. Many studies have found that after probiotic-induced fermentation, antioxidative, antiviral, antiinflammatory, and antitumor activities of vegetables increased [7–11]. Especially, *Lactobacillus plantarum*, a lactic acid bacterium with a vegetable origin, is a well-known probiotic that is commonly used as a starter culture for fermenting vegetables [12]. Many fermented foods containing *L*. *plantarum* as major strain have been reported to enhance health benefits during fermentation [12–15].

Despite these reports regarding the beneficial features of fermented vegetables, little has been elucidated regarding the causative factors behind the beneficial properties. More specifically, even less is known about how the metabolite changes during fermentation induce various health benefits. Previously, traditional fermented Korean vegetables such as soybeans [16] and kimchi [17] were studied for profiling fermentation-related metabolites, from which 41 [16] and 23 [17] metabolites were detected and identified, respectively. However, the small number of metabolites identified in those studies may be insufficient to either reflect or represent the general changes of metabolites during fermentation of vegetables.

Metabolomics is a study of comprehensive changes of metabolites that are caused either by or in living organisms [18]. Especially in case of fermentation processes, metabolites may also act as nutrients that directly affect growth of microorganisms and may be related to various health benefits arising from the fermented products [3,5]. To profile such metabolites, metabolomics is employed for unveiling metabolisms and identifying biomarkers for health benefits or diseases [18]. Thus, metabolomics can be used to interpret possible changes during the fermentation of vegetables by probiotics.

In this study, we hypothesized that various metabolites produced by probiotics fermenting prebiotic vegetables would induce health benefits. A total of 18 known prebiotic vegetables [7,19,20], such as tomato, cucumber, pear, apple, tangerine, water parsley, carrot, celery, onion, burdock, kale, spinach, aloe, chives, grape, jujube, cabbage, and perilla leaves, were mixed, and a representative probiotic microorganism, *L*. *plantarum* [12–15], was inoculated for fermenting the mixed vegetables. For analyzing the metabolite profile changes after fermentation of the mixed vegetables, gas chromatography/time-of-flight–mass spectrometry (GC/TOF–MS) was used. For assessing the health benefits from fermented mixed vegetables, antioxidative and antiinflammatory activities were tested by assays using 2,2-diphenyl-1-picrylhydrazyl (DPPH), nitric oxide (NO), interleukin-6 (IL-6), and tumor necrosis factor-α (TNF-α) cytokine in RAW 264.7 cells. To the best of our knowledge, this is the first report showing the relation between the changes in metabolite profiles and the enhancement of antioxidative and antiinflammatory activities of fermented vegetables.

## Materials and methods

### Mixed vegetable, a microbial strain, and fermentation conditions

To prepare fermented mixed vegetables, 18 different fruits and vegetables, namely, tomato, cucumber, pear, apple, tangerine, water parsley, carrot, celery, onion, burdock, kale, spinach, aloe, chives, grape, jujube, cabbage, and perilla leaves, were purchased at Hyundai Department Store (Seoul, South Korea). The fruits and vegetables were all produced in South Korea, of which cabbage and kale were organic, and the rest were conventionally grown. The fruits and vegetables were washed, sliced, and mixed with isomaltooligosaccharide, aloe extracts and starter culture. The composition (%, w/w) of the vegetable mixture is as follows; tomato (9.0%), cucumber (8.0%), pear (2.0%), apple (1.8%), tangerine (1.8%), water parsley (1.5%), carrot (1.5%), celery (1.5%), onion (1.5%), burdock (1.5%), kale (1.5%), spinach (1.5%), aloe (1.4%), chives (1.3%), grape (1.3%), jujube (1.3%), cabbage (1.0%), and perilla leaves (0.8%), isomaltooligosaccharide (24.0%), aloe extracts (36.0%, w/w), and the starter culture (0.005%). Three independent biological replicates of static cultivation were performed at 30°C with or without inoculation of *L*. *plantarum* PMO 08 for 72 h. The liquid portion in the mixture was obtained and used for CFU and pH measurement. Colony-forming units (CFUs) of total viable cells and total lactic acid bacteria were quantified by using plate count agar (Difco, Detroit, MI) and Bromo Cresol Purple (BCP) agar containing bromocresol purple (0.06 g/L) (Difco), respectively. To measure the pH of the mixture, a pH meter (Thermo Fisher Scientific, Waltham, MA) was used.

### Metabolite extraction from fermented mixed vegetables

Metabolites were extracted as previously described for metabolite extraction from plants with slight modification [21]. Mixed vegetables were completely ground to obtain vegetable juice, 400 μl of which was mixed with 1200 μl of acidified methanol (99.875% methanol acidified with 0.125% formic acid, v/v) for extracting metabolites. To completely disintegrate plant cell walls for effectively extracting metabolites, the vegetable juice in the acidified methanol mixture was thoroughly vortexed for 30 s, sonicated for 15 min at 20°C, centrifuged at 20,000 × *g* for 10 min, and filtered through a 0.2 μm filter. From the filtrate, 100 μl metabolites were obtained; they were completely vacuum-dried at room temperature and used for metabolite analyses. For each group, six replicates consisting of three independent biological replicates × two technical replicates from each biological replicate.

### High-performance liquid chromatographic analysis

A high-performance liquid chromatography (HPLC), equipped with a refractive index detector (Agilent 1100, Agilent Technologies, Waldbronn, Germany) and an Aminex HPX–87H organic acid column (Bio-Rad, Hercules, CA), was used for the quantification of lactic acid and acetic acid produced during fermentation. The mobile phase, 0.01 N H_2_SO_4_, was eluted at a constant flow rate of 0.5 ml/min at 65°C.

### GC/TOF–MS analysis of intracellular metabolites

For the identification and quantification of metabolites using GC/TOF–MS, methoximation and silylation were performed for derivatization of metabolites. For methoximation, 10 μl of 40 mg/ml methoxyamine hydrochloride in pyridine (Sigma-Aldrich, St. Louis, MO) was added to the metabolite samples, and the mixture was incubated at 30°C for 90 min. For silylation, 50 μl of *N*-methyl-*N*-trimethylsilyl-trifluoroacetamide (Fluka, Buchs, Switzerland) was added to the metabolite samples, and the mixture was incubated at 37°C for 30 min.

For accurate analysis of metabolites using the GC/TOF–MS, quality control was performed on a daily basis following the same protocol using the identical reagents and analytical instruments with two blank samples and four calibration curves samples consisting of 31 pure reference compounds such as alanine and pyruvate [22]. A mixture of fatty acid methyl esters including methyl forms of C8, C9, C10, C12, C14, C16, C18, C20, C22, C24, C26, C28, and C30 was added to the derivatized sample as retention index markers. For the identification and relative quantification of metabolites, an Agilent 7890B GC (Agilent Technologies) coupled with a Pegasus HT-TOF MS (LECO, St. Joseph, MI) was used. Derivatized metabolite samples (0.5 μl) were injected into the GC instrument equipped with an Rtx-5Sil MS column (30 m length, 0.25 mm inner diameter, and 0.25 μm film thickness; Restek, Bellefonte, PA) with an additional 10 m guard column, in splitless mode. The initial oven temperature was set at 50°C for 1 min, then ramped to 330°C at a rate of 20°C/min, and held for 5 min. Mass spectra in the range of 85–500 *m/z* were recorded at an acquisition rate of 10 spectra/s. The temperatures of the ion source and the transfer line of TOF–MS were 250°C and 280°C, respectively. The injected sample was ionized by electron impact at 70 eV. For accurate analysis, derivatization and analysis of an entire sample set were completed in one day.

### Data processing and statistical analyses for GC/TOF–MS raw data

For detection of peaks and deconvolution of mass spectra, the LECO ChromaTOF software (C version; LECO, St. Joseph, MI) was used for pre-processing the GC/TOF–MS raw data. The pre-processed data were further processed using an in-house library software, BinBase, for the identification of metabolites using the Fiehn library and the NIST library based on retention time and mass spectral similarities [23,24]. Peaks showing mass spectral similarity thresholds over 700 in comparison with authentic standards were regarded identical to their authentic standards. Intensities of the metabolites were reported as peak heights of their unique ion intensities. To treat missing values, the lowest background intensity was subtracted from the intensity of the quantified ion in its retention time region ± 5 s using MZmine software [24]. Raw data table was uploaded (S1 Table). Intensities of identified metabolites were normalized by volume of the vegetable extract. The normalized data were used in the statistical analyses, using partial least squares discriminant analysis (PLS-DA), hierarchical cluster analysis (HCA), Student’s *t*-test, Analysis of variance (ANOVA) and MetaMapp. PLS-DA was performed using the SIMCA-P+ software (version 12.0; Umetrics AB, Umea, Sweden). Student’s *t*-test and ANOVA were performed using the Statistica software (version 7.1; StatSoft, Tulsa, OK). HCA was performed using the MultiExperiment Viewer application [25]. The MetaMapp was performed using the MetaMapp and Cytoscape softwares [26].

### DPPH, nitric oxide, IL-6, and TNF-α assays

For the DPPH assay, 1.0 ml of 0.2 mM DPPH solution was added to 2 ml of each sample, the mixture was vortexed and incubated for 30 min. The absorbance of the mixture was measured at 517 nm, and the percentage difference between the absorbances before and after incubation was calculated. For the *in vitro* tests using RAW cells, 100 μl of RAW 264.7 cells (5 × 10^5^ cells/ml) in Dulbecco's modified eagle’s medium (GE Healthcare, Chicago, IL) were transferred into a 96-well plate. After 24 h incubation, the medium was removed, and serum-free medium containing 1 mg/ml of the ground fermented fruits and vegetable sample and 100 ng/ml lipopolysaccharides (LPS) were added to the cells. After incubation for 24 h, 50 μl of the mixture of cells and media was used for analyzing NO, IL-6, and TNF-α levels using a NO detection kit (iNtRON Biotechnology, Seongnam, South Korea), an IL-6 enzyme-linked immunosorbent assay (ELISA) kit (Enzo, Farmingdale, NY), and a TNF-α ELISA kit (Enzo), respectively. For positive and negative controls, LPS or blank controls were used.

## Results and discussion

### Profiles of bacterial growth, pH, and extracellular metabolites during fermentation

**Fig 1:**
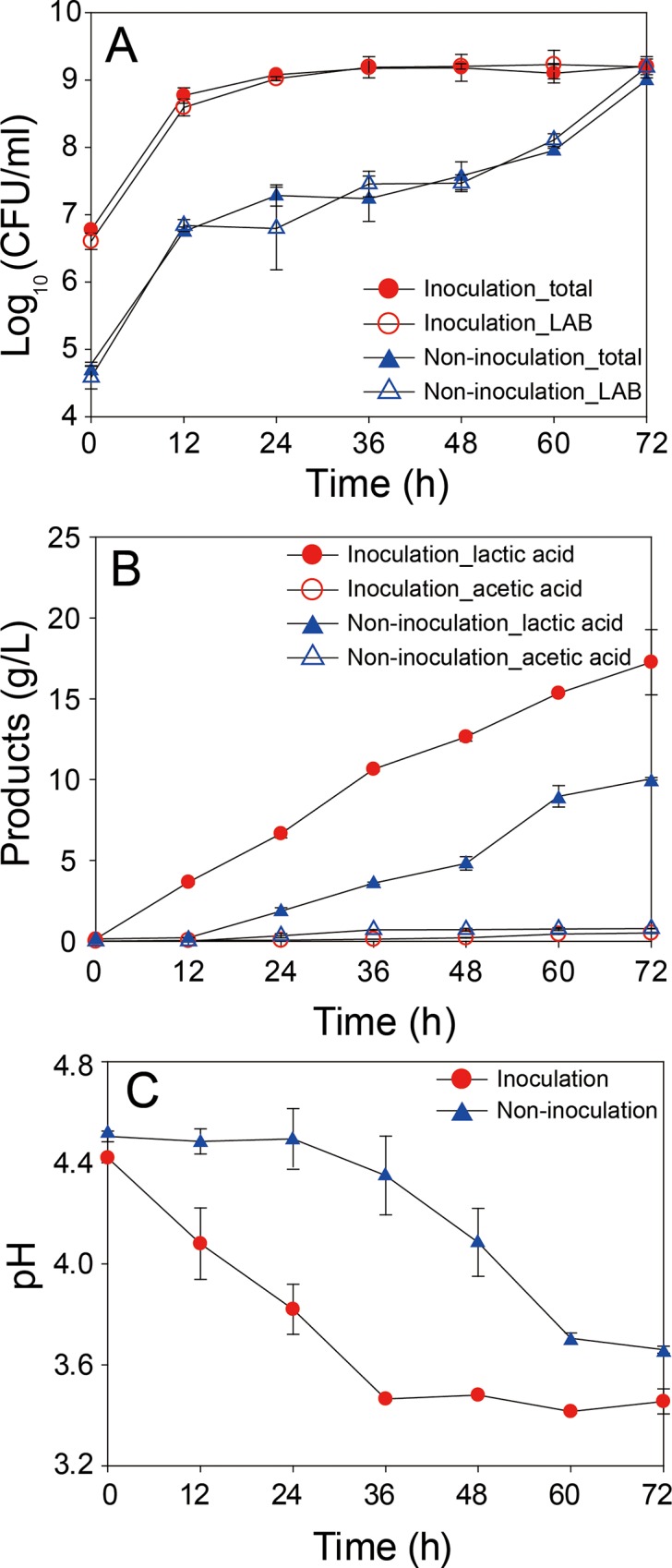
Comparison of fermentation profiles of the vegetable mixture. Colony forming units of total bacteria and lactic acid bacteria (A), concentration of lactic acid and acetic acid (B), and pH (C) are shown.

Growth profiles of total and lactic acid bacteria (Fig 1A), extracellular metabolites, namely, lactic acid and acetic acid (Fig 1B), and pH (Fig 1C) of mixed vegetables were investigated during fermentation (Fig 1). In the growth profiles, inoculated mixed vegetable samples showed much higher initial viable cell numbers (Fig 1A), cell growth rates (Fig 1A), and final lactic acid concentrations (Fig 1B) than the non-inoculated vegetable samples. However, cell growth in the inoculated samples, stopped at 24 h, but lactic acid was actively produced until 72 h (Fig 1A and 1B). On the other hand, the non-inoculated samples showed two distinct exponential phases: one between 0 and 12 h and the other between 60 and 72 h (Fig 1A). Interestingly, the non-inoculated samples showed much higher acetic acid concentrations than the inoculated samples (Fig 1B), even though the former had lower viable cell numbers.

The mixed vegetables might have contained a diverse microbial community, consisting of various microorganisms because they were not pre-sterilized. Therefore, *L*. *plantarum* inoculation as the starter culture could affect the composition of the microbial community and hence the concentration of products. In contrast, the non-inoculated samples, showing lower initial cell numbers, would be affected directly by the various microorganisms present initially in the community derived from soil. This difference in the composition of microbial community in the mixed vegetables could result in the differences observed in growth patterns, product yield, and final microbial compositions (Fig 1A and 1B). To obtain reproducible data, the microbial composition of mixed vegetables was controlled in the inoculated samples and used for the analyses of metabolome and health benefits.

### Identification of metabolites using GC/TOF–MS

To identify various metabolites in the mixed vegetable extracts, GC/TOF–MS was used. A total of 78 metabolites were detected and identified and comparatively quantified (S1 Table). Among the identified metabolites, the most frequently detected was a class of organic acids, which accounted for 28.2% of the total number of identified metabolites. Chemical classes of sugars and sugar alcohols, amino acids, fatty acids, amines, phosphates, and miscellaneous metabolites accounted for 23.1%, 20.5%, 14.1%, 5.1%, 5.1%, and 3.9%, respectively. The entire 78 metabolites identified by GC/TOF–MS in this study were used for statistical analyses of metabolite profiles of mixed vegetables.

### Overall changes of metabolites during fermentation of mixed vegetables

**Fig 2:**
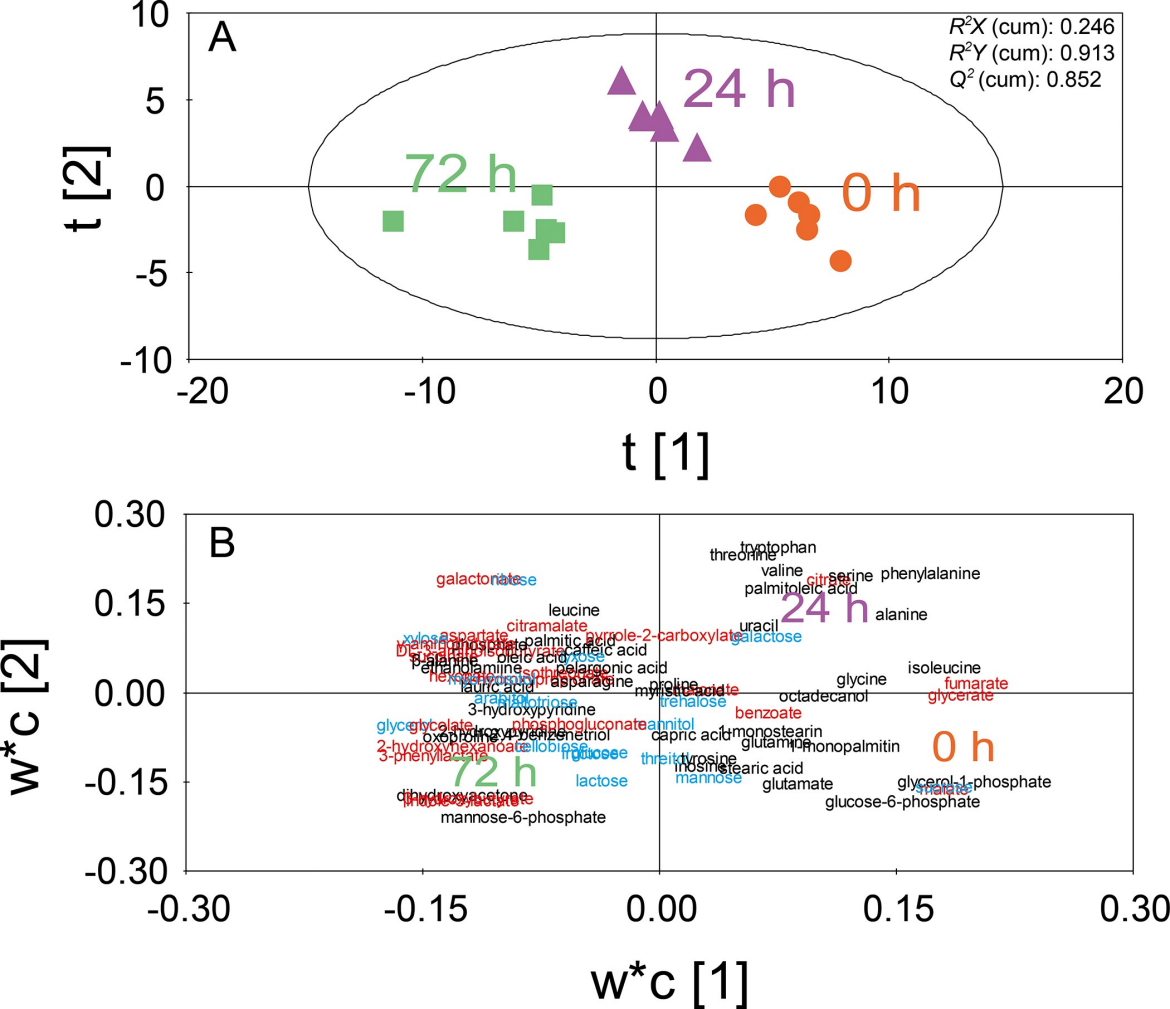
PLS-DA of 78 identified metabolites from mixed vegetables at 0 h, 24 h, and 72 h after inoculation of *Lactobacillus plantarum*. PLS-DA score plot (A) and PLS-DA loading plot (B) are shown. Colors represent chemical classes of metabolites (red: organic acids; blue: sugars and sugar alcohols; black: others).

In order to statistically compare the overall change of metabolite profiles during fermentation, PLS-DA analysis was performed (Fig 2). The score plot of PLS-DA analysis using two axes showed clear separation between the metabolite profiles of different fermentation times (0, 24, and 72 h) based on the t[1] axis (Fig 2A). Samples at 0 h, 24 h, and 72 h were located on the negative, center, and positive sides of the score plot, respectively. The PLS-DA model, using two axes, showed a high explanation capability with a cumulative *R*^*2*^*Y* of 0.913 and a high prediction capability with a cumulative *Q*^*2*^ of 0.852. To show the distribution tendency of metabolites, a loading plot of the PLS-DA model was created (Fig 2B). The VIP values and loading scores of the PLS-DA model are listed in S2 Table. The loading plot of PLS-DA analysis showed many metabolites located on the negative side where the samples fermented for 72 h were placed (Fig 2B). In contrast, fewer metabolites were located at the center and positive side of the loading plot, which corresponded to the samples fermented for 0 h and 24 h (Fig 2B). Overall, the PLS-DA score and loading plots showed time-dependent changes of the metabolite profiles (Fig 2). Intensity of most metabolites increased during fermentation, many of which occurred on the negative side of the loading plot. Interestingly, among the metabolites that increased in abundance, organic acids, sugars, and sugar alcohols were predominant (Fig 2B). These metabolites may have been either newly synthesized or derived from the degradation of high molecular weight polymers from vegetables during fermentation.

### Time-dependent changes of metabolite abundance during fermentation

**Fig 3:**
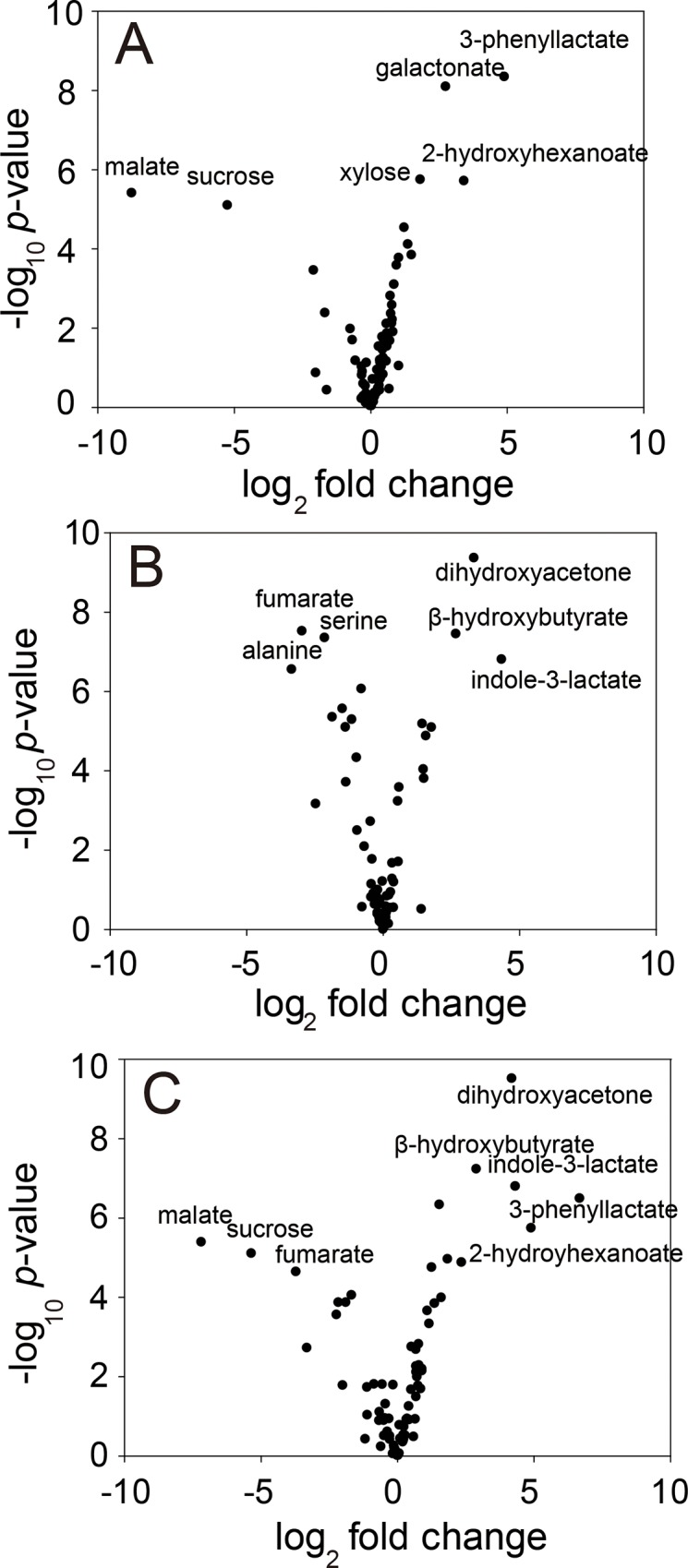
Volcano plots of 78 primary metabolites based on Student’s *t*-test. Comparison between 0 h and 24 h (A), 24 h and 72 h (B), and 0 h and 72 h (C) are shown.

To investigate the changes in metabolite abundance in more details, a volcano plot based on Student’s *t*-test was constructed (Fig 3). Comparing the samples from 0 h with those from 24 h, malate and sucrose were found depleted, and various organic acids such as 3-phenyllactate and 2-hydroxyhexanoate, monomeric sugars such as xylose, and amino acids such as β-alanine and tryptophan accumulated after 24 h (Fig 3A and S3 Table). During this period, microorganisms might have adapted to the fermentation environment, and actively grown using sucrose and malate. Microorganisms could synthesize various amino acids such as β-alanine and tryptophan for protein synthesis and cell growth, and could produce various organic acids including lactic acid, 3-phenyllactate, and 2-hydroxybutyrate. In this period, levels of various monomeric sugars also increased, which collectively indicated that the increased sugars probably resulted from the enzymatic degradation of polymers originating from either microorganisms [27] or vegetables [28].

Upon comparison between the samples at 24 h and 72 h, various amino acids such as alanine, serine, and phenylalanine and few organic acids such as citrate, glycerate, and fumarate were found to have significantly decreased (Fig 3B and S4 Table). Especially, citrate, which was analyzed to be the main component of the mixed vegetables before fermentation in this experiment, decreased remarkably during fermentation. In contrast, organic acids such as indole-3-lactate, β-hydroxybutyrate, and 3-phenyllactate and sugars such as dihydroxyacetone and glycerol significantly increased. It is likely that, during the fermentation period between 24 h and 72 h, microorganisms might have entered the stationary phase, producing various organic acids including lactic acid and low molecular weight sugars, rather than amino acids essential for cell growth. Citrate might have been utilized by microorganisms as an energy source [29].

**Fig 4:**
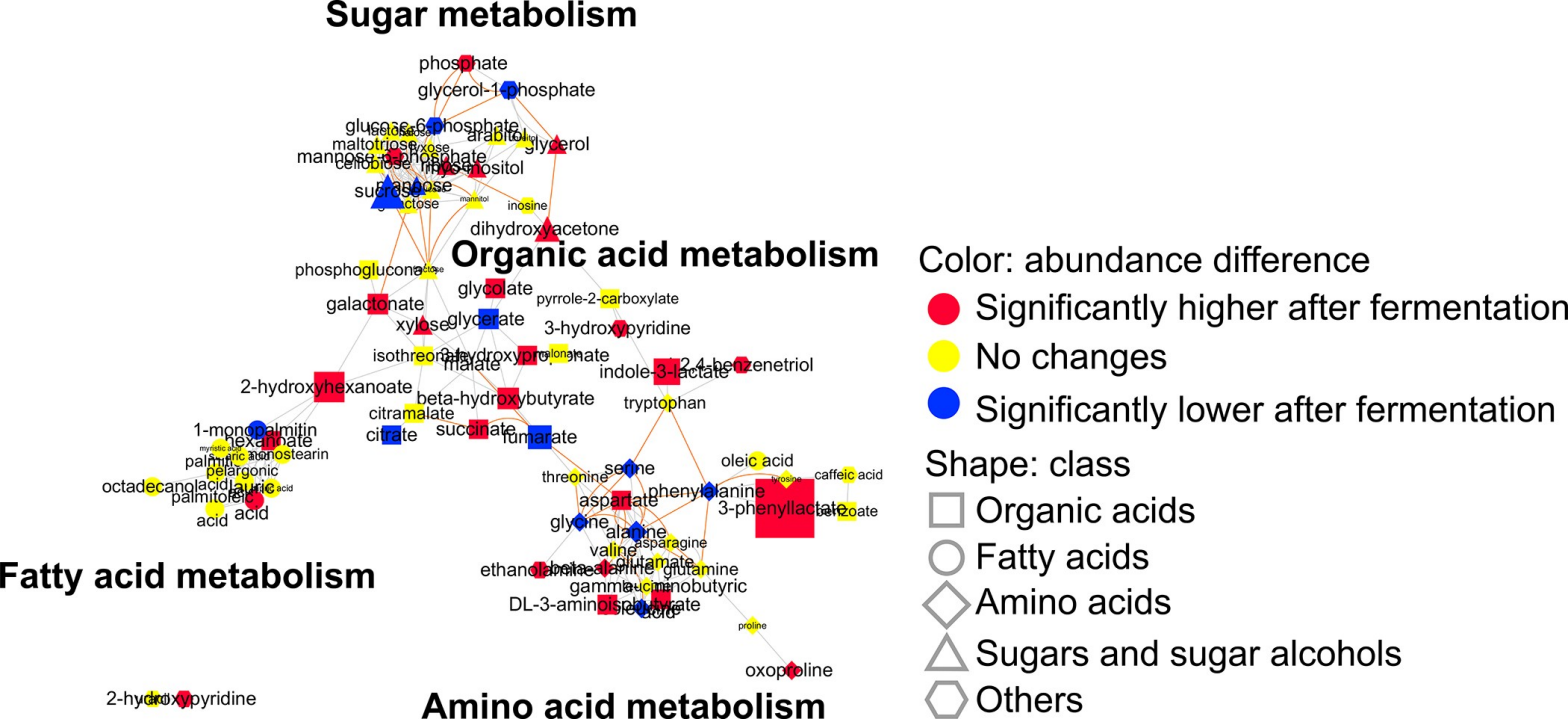
MetaMapp analysis for mapping 78 primary metabolites. Classes of metabolites are represented by shape. Significant changes of metabolites are represented by color (*p* < 0.05). Magnitudes of fold changes are represented by sizes of symbols and labels. Biochemical and structural similarities are represented by purple and gray edges, respectively.

**Fig 5:**
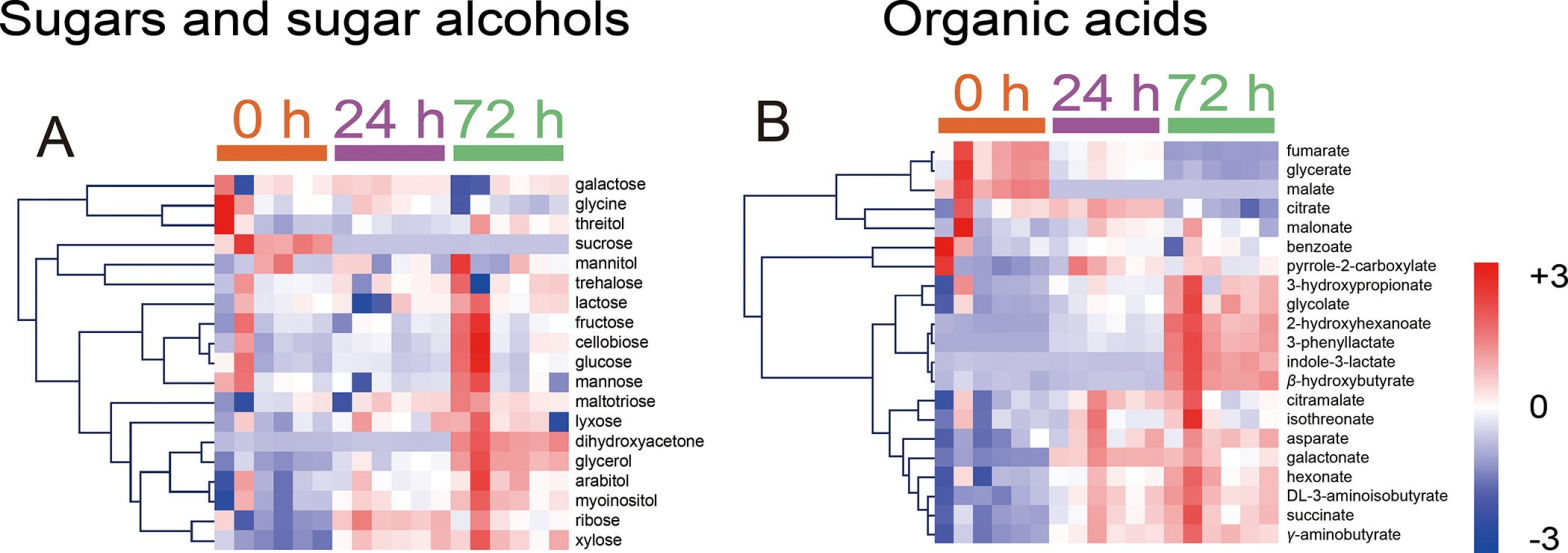
HCA of classes of sugars, sugar alcohols, and organic acids. HCA plots for sugars and sugar alcohols (A) and organic acids (B) are shown. HCA correlations were based on the Pearson correlation.

To compare the changes in metabolites before and after fermentation, samples at 0 and 72 h after inoculation were analyzed (Fig 3C and S5 Table). To show the changes in metabolite abundance before and after fermentation, based on metabolic pathways and structural similarities of metabolites, the MetaMapp analysis was performed based on the results of *t*-test between 0 h and 72 h (Fig 4). Malate and sucrose, the two main components of mixed vegetables in this study, significantly decreased after fermentation. In contrast, various organic acids such as 3-phenyllactate, 2-hydroxyhexanoate, and indole-3-lactate and low molecular weight sugars such as dihydroxyacetone, xylose, and ribose significantly increased. During the entire fermentation process, increases of various sugars and organic acids were evident. To demonstrate the time-dependent changes in the concentration of sugars and organic acids, ANOVA was performed between 0 h, 24 h, and 72 h. A total of 7 sugars and sugar alcohols (S6 Table), and 17 organic acids were significantly changed during the fermentation (S7 Table). To show time-dependent significant increase of independent sugars, sugar alcohols, and organic acids, the HCA analysis based on the Pearson correlation was performed (Fig 5). The HCA analysis showed time-dependent increase of independent sugars, sugar alcohols (Fig 5A), and organic acids (Fig 5B).

Taken together, enzymes of microorganisms [27] and vegetables [28] might have degraded polymeric substances into various small molecules, mainly sugars or organic acids. Lactic acid bacteria might have utilized these small molecules, such as sucrose, malate, and citrate, as energy sources for the production of various organic acids such as lactic acid and 3-phennyllactate [29–31]. The increased levels of sugars and organic acids, during fermentation, might have antioxidative and antiinflammatory activities that could contribute to various health benefits in fermented vegetables.

### Antioxidative and antiinflammatory activities of mixed vegetables

**Fig 6:**
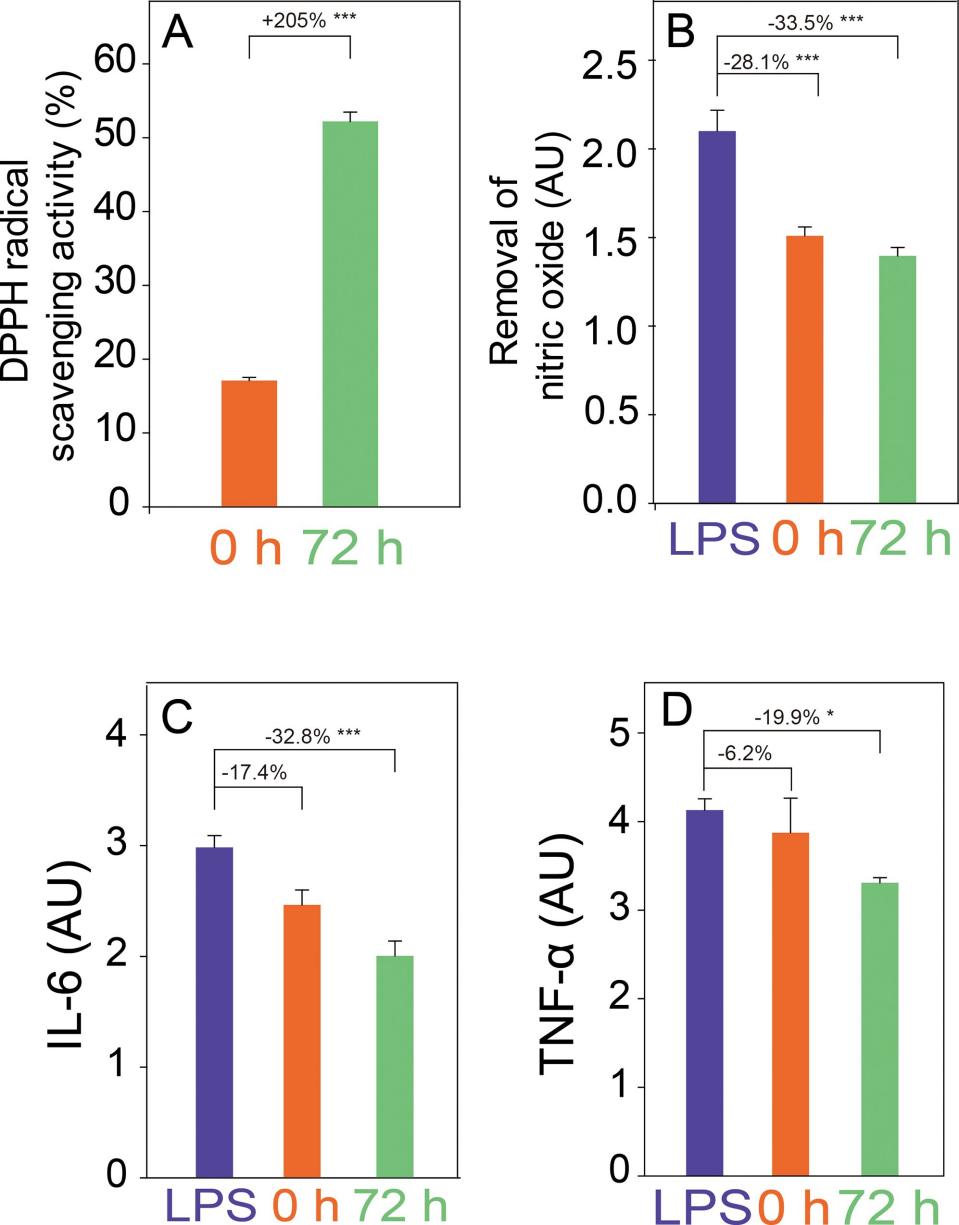
Antioxidative and antiinflammatory activities of mixed vegetables before and after fermentation by *Lactobacillus plantarum*. DPPH analysis (A), inhibition of nitric oxide (B), production of IL-6 (C), and TNF-α (D) in raw 264.7 cells are shown (*p < 0.05; ***p < 0.01).

To determine whether the health benefits of mixed vegetables increase post fermentation, their antioxidative and antiinflammatory activities were analyzed before and after fermentation (Fig 6). The DPPH and NO assays of RAW cells revealed radical scavenging activity of fermented and non-fermented mixed vegetables (Fig 6A and 6B) although the activity significantly increased after fermentation. The IL-6 and TNF-α levels of RAW cells, treated with mixed vegetables, were also analyzed (Fig 6C and 6D). Treatment with the fermented mixed vegetables showed significant decreases in IL-6 and TNF-α levels. Taken together, it is evident that the antioxidative and antiinflammatory activities of the mixed vegetables significantly increased after fermentation.

Increased antioxidative and antiinflammatory activities in the fermented fruits and vegetables are directly related to the increased metabolites, especially organic acids. Various organic acids are known to possess antioxidative and antiinflammatory activities [32]. As seen earlier in the metabolite analyses, the significant increase of various organic acids after fermentation were evident in this study (Fig 5B). Therefore, it is possible to relate the increased antioxidative and antiinflammatory activities (Fig 6) of the fermented vegetables to the associated increase of various organic acids in the fermented vegetables (Fig 5B). Among the significantly increased organic acids, lactate [33], 3-phenyllactate [34], indole-3-lactate [35], β-hydroxybutyrate [36], and γ-aminobutyrate [37] are well-known scavengers of reactive oxygen species with high antioxidative activity. In addition, lactate [38], indole-3-lactate [39], β-hydroxybutyrate [40], γ-aminobutyrate [41, 42], and glycerol [43], which were found to have significantly increased after fermentation, in this study, are known to be antiinflammatory substances. Moreover, health benefits of fermented vegetables may not be limited to the antioxidative and antiinflammatory activities explored in this study. Among the significantly increased metabolites after fermentation, γ-aminobutyrate [44], β-alanine [45], and succinate [46] were also reported as antitumor substances. In particular, these metabolites are known to enhance health benefits through synergic effects when they are in food as a mixture rather than as a single metabolite [47–49]. Therefore, these metabolites in the fermented vegetables could also render additional health benefits. To provide useful information for future studies of fermented mixed vegetables using *L*. *plantarum*, raw metabolite analysis data have been uploaded (S1 Table). However, our presumption that the increased health benefits are due to metabolites produced by *L*. *plantarum* has a limitation of a lack of experimental validation. This can be verified by further studies such as applying metabolomic approaches to the fermented mixed vegetables before and after inoculation of *L*. *plantarum*.

## Conclusions

In this study, a mixture of 18 different vegetables was fermented with *L*. *plantarum*. Significantly increased abundance of metabolites from primary metabolism, along with increased health benefits post fermentation, was observed in this study. During the fermentation of mixed vegetables, enzymes from both microorganisms and vegetables have degraded large molecules into small ones, which could have been utilized initially by the inoculated lactic acid bacteria as nutrients for their growth, producing various amino acids and organic acids. After growth completion, the lactic acid bacteria have produced much more organic acids including antioxidative (e.g. lactate, 3-phenyllactate, indole-3-lactate, β-hydroxybutyrate, and γ-aminobutyrate), antiinflammatory (e.g. lactate, indole-3-lactate, β-hydroxybutyrate, γ-aminobutyrate, and glycerol), and antitumor agents (e.g. γ-aminobutyrate, β-alanine, and succinate), resulting in increased health benefits of the fermented mixed vegetables. Our omics data and biological interpretation could be used to assist to unveil health benefits of vegetables fermented with lactic acid bacteria.

## Supporting information

S1 Table Metabolome data analyzed by GC/TOF–MS. (XLSX)

S2 Table VIP values and loading scores of PLS-DA using two axes. (XLSX)

S3 Table *t*-Test comparison between 0 h and 24 h. (XLSX)

S4 Table *t*-Test comparison between 24 h and 72 h. (XLSX)

S5 Table *t*-Test comparison between 0 h and 72 h. (XLSX)

S6 Table Comparison of sugars and sugar alcohols between 0 h, 24 h, and 72 h using ANOVA. (XLSX)

S7 Table Comparison of organic acids between 0 h, 24 h, and 72 h using ANOVA. (XLSX)
